# A case-control collapsing analysis identifies epilepsy genes implicated in trio sequencing studies focused on *de novo* mutations

**DOI:** 10.1371/journal.pgen.1007104

**Published:** 2017-11-29

**Authors:** Xiaolin Zhu, Raghavendra Padmanabhan, Brett Copeland, Joshua Bridgers, Zhong Ren, Sitharthan Kamalakaran, Ailbhe O'Driscoll-Collins, Samuel F. Berkovic, Ingrid E. Scheffer, Annapurna Poduri, Davide Mei, Renzo Guerrini, Daniel H. Lowenstein, Andrew S. Allen, Erin L. Heinzen, David B. Goldstein

**Affiliations:** 1 Institute for Genomic Medicine, Columbia University Medical Center, New York, NY, United States of America; 2 Department of Medicine, Royal College of Surgeons in Ireland, St Stephen's Green, Dublin, Ireland; 3 Epilepsy Research Centre, Department of Medicine, University of Melbourne at Austin Health, Heidelberg, Australia; 4 Florey Institute for Neuroscience and Mental Health, University of Melbourne, Heidelberg, Australia; 5 Departments of Paediatrics and Neurology, Royal Children's Hospital, University of Melbourne, Melbourne, Australia; 6 Epilepsy Genetics Program and Department of Neurology, Harvard Medical School, Boston, MA, United States of America; 7 Pediatric Neurology Unit and Laboratories, Meyer Children’s Hospital, University of Florence, Florence, Italy; 8 IRCCS Stella Maris Foundation, Pisa, Italy; 9 Department of Neurology, University of California, San Francisco, San Francisco, California, United States of America; 10 Department of Biostatistics and Bioinformatics, Duke University, Durham, North Carolina, United States of America; HudsonAlpha Institute for Biotechnology, UNITED STATES

## Abstract

Trio exome sequencing has been successful in identifying genes with *de novo* mutations (DNMs) causing epileptic encephalopathy (EE) and other neurodevelopmental disorders. Here, we evaluate how well a case-control collapsing analysis recovers genes causing dominant forms of EE originally implicated by DNM analysis. We performed a genome-wide search for an enrichment of "qualifying variants" in protein-coding genes in 488 unrelated cases compared to 12,151 unrelated controls. These "qualifying variants" were selected to be extremely rare variants predicted to functionally impact the protein to enrich for likely pathogenic variants. Despite modest sample size, three known EE genes (*KCNT1*, *SCN2A*, and *STXBP1*) achieved genome-wide significance (p<2.68×10^−6^). In addition, six of the 10 most significantly associated genes are known EE genes, and the majority of the known EE genes (17 out of 25) originally implicated in trio sequencing are nominally significant (p<0.05), a proportion significantly higher than the expected (Fisher’s exact p = 2.33×10^−17^). Our results indicate that a case-control collapsing analysis can identify several of the EE genes originally implicated in trio sequencing studies, and clearly show that additional genes would be implicated with larger sample sizes. The case-control analysis not only makes discovery easier and more economical in early onset disorders, particularly when large cohorts are available, but also supports the use of this approach to identify genes in diseases that present later in life when parents are not readily available.

## Author summary

Trio exome sequencing and *de novo* mutation (DNM) analysis has been the main approach to discovering genes responsible for severe sporadic disorders, including a range of neurodevelopmental disorders. This approach requires sequencing parents, identifying DNMs from trio sequence data, and comparing the observed rate of DNMs to the expected. In this study, we adopted a case-control design, performed a gene-based collapsing analysis, and rediscovered several of the epileptic encephalopathy (EE) genes originally implicated by DNM analysis of EE trios. Our collapsing analysis focused on ultra-rare, highly impactful variants (“qualifying variants”) by filtering against large-scale population datasets, and this approach revealed that most of the standing variation can be filtered out and DNMs are enriched in “qualifying variants”. Our study suggests that a case-control analysis approach can be used to identify disease genes with causal mutations that are predominantly *de novo* in place of trio-based analysis methods. This offers an efficient and cost effective alternative approach when large-scale trio sequencing is not possible.

## Introduction

One of the most important recent developments in human genomics is the use of a trio sequencing paradigm to implicate new disease genes in sporadic disease by evaluating patterns of *de novo* mutations (DNMs). This framework compares the observed pattern of DNMs in probands to the expected based on the size of the protein-coding sequence and the estimated tri-nucleotide mutation rate[1], and has implicated scores of genes conferring risk of epilepsy[2, 3], intellectual disability[4–6], autism[7–10], and other neurodevelopmental conditions[4]. This approach is costly because of the need to sequence complete trios and often is not practical or possible for conditions that present after childhood where parents may not be available for sequencing. Moreover, a precise estimate of mutation rate is not available for small insertion/deletions (indels)[1], limiting the ability to assess the significance of genes harboring *de novo* indels.

In parallel to these developments, collapsing analyses, which typically compare the burden of rare, presumably deleterious variants gene by gene in cases versus controls, have proven increasingly successful in implicating diseases genes, for example in amyotrophic lateral sclerosis[11, 12], idiopathic pulmonary fibrosis[13, 14], and monogenic disorders[15]. Surprisingly, however, it has not yet been assessed whether the collapsing framework can identify the genes implicated by analysis of trio sequencing data. We addressed this question by implementing a genome-wide gene-based collapsing analysis using whole exome sequencing (WES) data generated from 488 epileptic encephalopathy (EE) patients, including those previously analyzed using the trio-based DNM analysis framework, and a large cohort of unrelated control individuals to assess the efficacy of case-control analysis to identify disease genes implicated by DNM analysis for EE. Strikingly, despite a modest sample size, we identified three known EE genes achieving genome-wide significance (p<2.68×10^−6^), and found that the majority of the known EE genes (17 out of 25) originally implicated in trio sequencing are nominally significant (p<0.05). While not all known EE genes reached genome-wide significance, the significant enrichment of known genes among nominally significant p-values genome-wide suggests that with larger samples sizes many of these genes will reach p-values that will exceed that threshold. Collectively, our results show that collapsing analysis can effectively implicate genes carrying causal DNMs, and trio sequencing is not the only effective strategy for gene discovery even in genes that confer risk largely due to DNMs. We argue that the fundamental reason for this is that existing filtering strategies are increasingly accurate in identifying very young mutations including those that are *de novo* in the proband.

## Results and discussion

**Fig 1:**
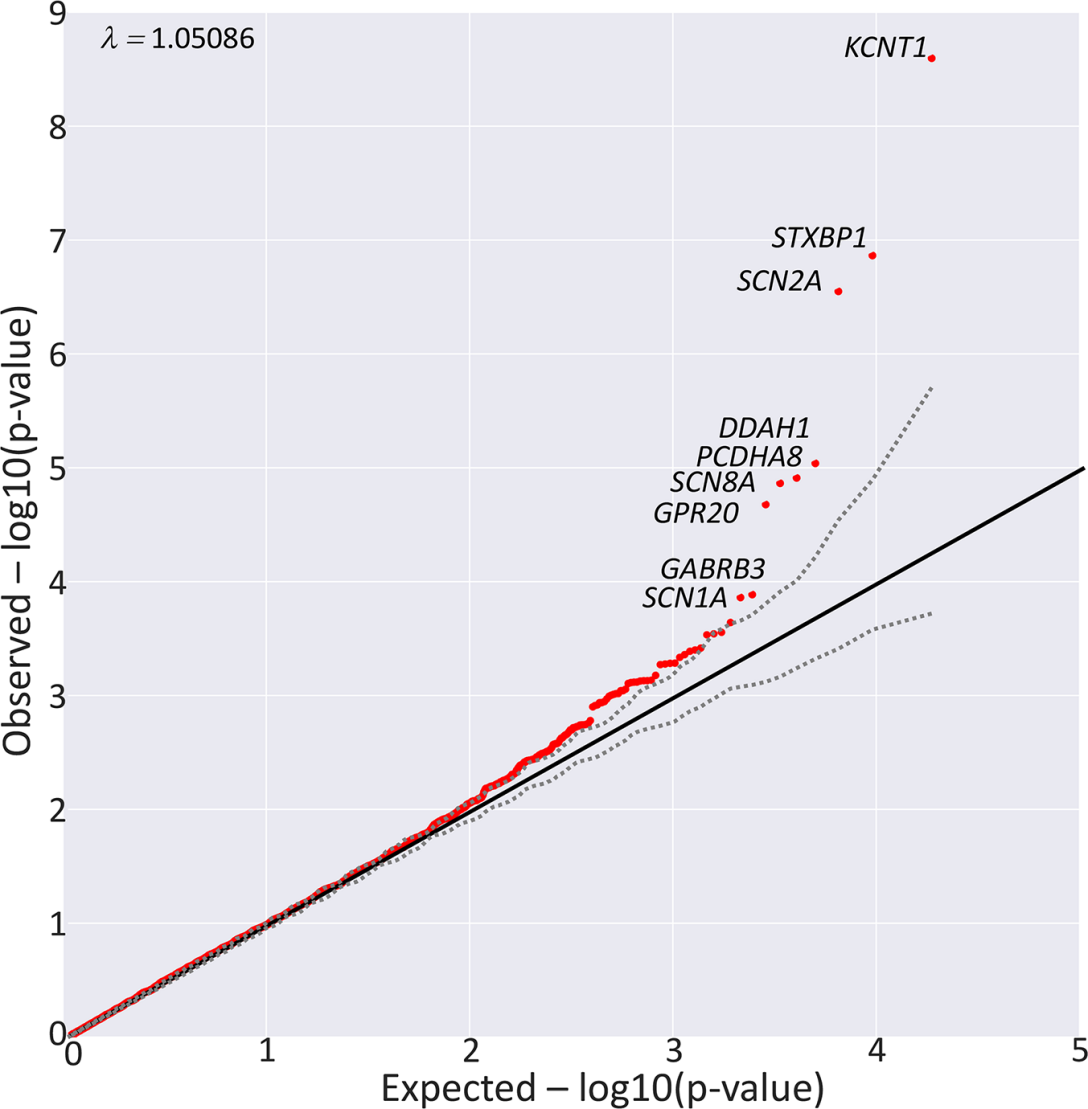
A. Quantile-quantile (QQ) plot for genome-wide gene-based collapsing analysis. The Y-axis represents -log10 of observed (red) p-values (sorted) evaluated in the logistic regression adjusting for the total number of “ultra-rare” synonymous variants per individual; the X-axis represents -log10 of expected p-values (sorted) evaluated in the same logistic regression model with permutation (“BiasedUrn”). The dashed grey lines indicate permutation-based 95% confidence intervals.

The collapsing analysis compared a total of 488 cases with 12,151 controls (S1 Fig). Three genes (Fig 1, Table 1, S1 Table, and S2 Fig), *KCNT1*, *SCN2A* and *STXBP1*, showed enrichment of qualifying variants in EE patients and achieved genome-wide significance (p<2.68×10^−6^). No other genes were found to be genome-wide significant by both Fisher’s exact test and logistic regression p-values, but 17 of the 25 genes (68%, including the three above) known to be associated with dominant EE (https://www.omim.org/phenotypicSeries/PS308350) were nominally significant (logistic regression p<0.05) in this dataset, all showing enrichment of qualifying variants in EE patients (Table 1). This is in contrast to the total of 885 nominally significant (logistic regression p<0.05) genes out of all the 18,503 genes tested (Fisher’s exact p = 2.33×10^−17^). We used a hypergeometric test to assess whether these 25 known dominant EE genes tend to have lower p-values in our case-control gene-based collapsing analysis compared with the rest of the genome. Specifically, at each observed ranking of the 25 epilepsy genes (based on logistic regression p-values), we performed a hypergeometric test to assess whether there were more epilepsy genes at this ranking, or lower, than one would expect if the ranks were randomly assigned to all 18,503 genes tested (Table 1). There was a consistent pattern that known dominant EE genes tended to have smaller p-values in our dataset (Table 1).

**Table 1:** Association results for the 25 genes known to cause dominant forms of epileptic encephalopathy.

Logistic P Rank	Gene	Case (Trios)^c^	Case Frequency	Control^d^	Control Frequency	Collapsing Logistic P^e^	Collapsing Fisher's Exact P	Probability of seeing this many (or more) known epilepsy genes at this ranking^f^	Case *de novo*^g^	Case inherited^g^
1	*KCNT1*^*a*^	10 (4)	0.0205	14	0.0012	2.53E-09	8.1E-09	1.35E-03	2^h^	1^h^
2	*STXBP1*^*a*^	6 (6)	0.0123	4	0.0003	1.37E-07	5.9E-07	1.75E-06	6	0
3	*SCN2A*^*a*^	8 (7)	0.0164	14	0.0012	2.82E-07	9.3E-07	2.18E-09	4	3
6	*SCN8A*^*b*^	8 (8)	0.0164	26	0.0021	1.37E-05	3.5E-05	3.88E-11	4	4
8	*GABRB3*^*b*^	3 (2)	0.0061	2	0.0002	1.30E-04	0.00054	1.64E-13	2	0
9	*SCN1A*^*b*^	8 (8)	0.0164	36	0.0030	1.38E-04	0.00024	2.66E-16	7	1
14	*GRIN2B*^*b*^	5 (5)	0.0102	16	0.0013	3.83E-04	0.001	1.11E-17	2	3
18	*GABRA1*^*b*^	3 (3)	0.0061	4	0.0003	4.62E-04	0.0018	1.38E-19	2	1
23	*DNM1*^*b*^	5 (5)	0.0102	18	0.0015	6.65E-04	0.0016	2.36E-21	5	0
41	*KCNQ2*^*b*^	3 (3)	0.0061	6	0.0005	1.13E-03	0.004	2.77E-21	3	0
42	*SPTAN1*^*b*^	7 (7)	0.0143	40	0.0033	1.15E-03	0.002	8.59E-24	1	6
136	*HCN1*^*b*^	2 (1)	0.0041	4	0.0003	6.43E-03	0.0201	7.26E-20	0	1
317	*ALG13*^*b*^	1 (1)	0.0020	1	0.0001	0.017	0.0757	3.71E-17	1	0
353	*GABRB1*^*b*^	2 (2)	0.0041	7	0.0006	0.019	0.0447	2.41E-18	1	1
402	*GNAO1*^*b*^	2 (2)	0.0041	9	0.0007	0.023	0.065	2.35E-19	2	0
409	*CDKL5*^*b*^	3 (3)	0.0061	17	0.0014	0.023	0.04	4.14E-21	3	0
737	*SLC35A2*^*b*^	1 (1)	0.0020	3	0.0002	0.045	0.1457	1.07E-18	1	0
1411	*SIK1*	1 (0)	0.0020	5	0.0004	0.086	0.2105	1.97E-15	N/A	N/A
1944	*GRIN2D*	1 (1)	0.0020	7	0.0006	0.124	0.2703	2.24E-14	0	1
3232	*KCNB1*	1 (1)	0.0020	11	0.0009	0.228	0.3767	1.43E-11	0^h^	0^h^
3732	*CACNA1A*	2 (2)	0.0041	28	0.0023	0.277	0.3234	1.29E-11	1	1
3755	*PCDH19*	1 (1)	0.0020	13	0.0011	0.280	0.4239	6.65E-13	0	1
6110	*EEF1A2*	0 (0)	0	3	2.47E-04	0.453	1	1.17E-09	N/A	N/A
11309	*KCNA2*	0 (0)	0	7	5.76E-04	0.730	1	7.57E-05	N/A	N/A
13606	*SLC1A2*	0 (0)	0	10	8.23E-04	0.847	1	4.57E-04	N/A	N/A

^a^ Genome-wide significant (both logistic regression and Fisher’s exact p-values <2.68×10^−6^).

^b^ Nominally significant (logistic regression p-value <0.05).

^c^ Number of cases carrying at least one qualifying variant in the gene (number of complete trios sequenced).

^d^ Number of controls carrying at least one qualifying variant in the gene.

^e^ Adjusted for the total number of "ultra-rare" synonymous variants per individual (see Methods).

^f^ Probabilty of observing *x*, or more, known epilepsy genes at ranking *r* if one were to randomly draw *r* genes at random from a collection of 18,503 genes with 25 being known epilepsy genes. Calculation by upper tail of hypergeometric distribution characterizing sampling without replacement from a collection 25 known epilepsy genes and 18,478 non-epilepsy genes.

^g^ In cases with trio WES data.

^h^ One trio without DNA available for all three for Sanger validation.

In the 25 genes known to cause dominant forms of EE, 74 of the 488 cases (15.16%) had at least one qualifying variant, compared to 302 of the 12,151 controls (2.49%, Fisher’s exact p = 1.95×10^−32^). Among the 64 of the 74 cases with trio WES data, a total of 73 qualifying variants were found in these 25 EE genes, and 47 of these qualifying variants (64.4%) were confirmed to be *de novo* in our previous DNM analyses (Table 1 and S2 Table), including all the qualifying variants in *STXBP1* (n = 6), *DNM1* (n = 5), *KCNQ2* (n = 3), *GNAO1* (n = 2), *CDKL5* (n = 3), *ALG13* (n = 1) and *SLC35A2* (n = 1) identified in the 488 cases (no inherited qualifying variant was observed in these genes in all cases; Table 1).

Comparing 488 EE cases and 12,151 controls using a gene-based collapsing analysis of “qualifying variants”, we successfully identified three known EE genes at genome-wide significance level. In addition, known EE genes were found to have smaller than expected association p-values compared with the rest of the genome. We showed that DNMs contributed to the majority of qualifying variants in the 25 known dominant EE genes identified in cases, and in several genes they accounted for all of them. As most of these 25 EE genes are originally implicated by sequencing trios and analyzing DNMs, our results clearly demonstrate the efficacy of case-control gene-based collapsing analysis to identify genes without spending effort specifically ascertaining DNMs by sequencing trios.

Several factors affect the power of case-control gene-based collapsing analysis, including locus heterogeneity, penetrance, and how “qualifying variants” are defined as a class to represent the properties of *bona fide* pathogenic mutations. Because most if not all known EE-causing mutations are not observed in ExAC, we required the qualifying variants to be absent in ExAC. Remarkably, because of the large sample size of ExAC, most standing variation is essentially filtered out (except mutations arising in recent generations, including DNMs), and indeed 64.4% of the qualifying variants in the 25 known EE genes are confirmed to be *de novo* in 64 cases, thus recovering many of the EE genes originally implicated by DNM analysis. Notably, all the six *STXBP1* and five *DNM1* qualifying variants in cases are *de novo*, highlighting the power of using ExAC to filter out standing variation. However, even at the sample size of ExAC, where widespread mutational recurrence is observed[16], background variation in controls may still prevent a gene that is securely implicated in DNM analysis from reaching genome-wide significance in case-control analysis. For example, in *DNM1*, even with five qualifying variants (all DNMs) in unrelated cases, there are 18 qualifying variants in controls unfiltered by ExAC. These 18 qualifying variants may be private but not DNMs, and may be further filtered out by a larger and more genetically diverse control datasets. Indeed, although most genes known to cause EE (and other neurodevelopmental disorders) are intolerant to standing functional variation[17], implying a lower rate of background variation than the genomic average, our empirical data shows considerable variability in the frequency of qualifying controls across the 25 EE genes (Table 1). Versions of collapsing that focus on subregions of genes will likely allow finer discriminations amongst pathogenic variants and background variation.

As a class, disease-causing DNMs clearly represent the extreme of rare variation by typically not being able to pass even one generation due to extremely strong negative selection. However, this does not mean every DNM identified in an individual is pathogenic, and there are DNMs presenting as standing variation in human population datasets like ExAC and these DNMs are unlikely to be pathogenic[18]. By focusing on qualifying variants absent in ExAC, such presumably benign DNMs can be excluded from collapsing analysis. Conversely, if a pathogenic variant is inherited and the parent is not known to be affected (e.g., due to incomplete penetrance or variable phenotype), it would not be identified in trio-based analyses focused on DNMs but may be captured in case-control analyses.

The DNM analysis framework typically compares observed rate of DNMs in cases with expectation relying on estimates of the mutability of genes since very large populations of control trios are not available for direct comparisons. Precisely estimating mutation rate across the human genome is difficult and the current DNM analysis framework cannot effectively accommodate indels well due to lack of accurate estimations of mutation rate for this class of variants. However, case-control analysis directly compares the pattern of qualifying variants empirically observed in both cases and controls and is not affected by mutation rate estimates.

When a disease gene is securely implicated using a case-control framework, caution is needed to interpret the causality of qualifying variants identified in that gene. Importantly, an excess of qualifying variants in cases versus controls does not imply all qualifying variants in cases are pathogenic or all qualifying variants in controls are benign. Instead, interpretation should be performed per variant per individual after the case-control association testing is performed. Certainly, for an individual case, knowledge of whether a variant is *de novo* or not remains an important consideration in diagnostic interpretation[19]. However, our work clearly shows that a collapsing analysis using only probands can also discover genes that cause disease due to DNMs. This not only makes discovery easier and more economical in early onset disorders, but opens up the possibility of identifying genes that carry causal DNMs in diseases that present later in life when parents are not readily available. These results have clear implications for discovery strategies in a range of different genetic diseases.

## Materials and methods

### Subjects and sequencing

We started with WES or whole genome sequencing (WGS) data generated from 496 cases selected from several genetic studies of EE and 12,916 controls selected from other studies and not known to have neurodevelopmental, neuropsychiatric, or severe pediatric diseases. The cases were originally recruited and studied by groups including the Epi4K Consortium, the Epilepsy Phenome Genome Project (EPGP), the Epilepsy Genetics Initiative (EGI)—a signature program of Citizens United for Research in Epilepsy (CURE), and EuroEPINOMICS-RES Consortium.

Written informed consent was collected at the time of recruitment at each of the clinical sites. Patient collection and sharing of anonymized specimens for research was approved by site-specific Institutional Review Boards and ethic committees. Details of the IRB and approval numbers are available from S3 Table.

To maximize sample size, both cases and controls included individuals with diverse ancestries including African, Caucasian, East Asian, Hispanic, Middle Eastern, and South Asian. After relatedness check and principal component analysis, a total of 488 cases and 12,151 controls remained for association analysis, and 75.6% of cases (n = 369, S4 Table) had been analyzed previously in trio or single-patient interpretation analyses.

Sequencing was performed at multiple sites (S2 Table). All data starting from either FASTQ or BAM files were processed through the alignment and annotation pipeline at the Institute for Genomic Medicine at Columbia University Medical Center (formerly Center for Human Genome Variation at Duke University). Case (S2 Table) and control samples were sequenced after exome capture using a variety of technologies (Agilent Clinical Research Exome, IDT xGen Exome Research Panel V1.0, Illumina Nextera Rapid Capture—Expanded Exome [62MB], SeqCap EZ Exome v2, SeqCap EZ Exome v3, SeqCap EZ MedExome, SureSelect Human All Exon - 50MB, SureSelect Human All Exon - 65MB, SureSelect Human All Exon V4, SureSelect Human All Exon V4 - 50MB, SureSelect Human All Exon V4 + UTR, SureSelect Human All Exon V5, SureSelect Human All Exon V5 + UTR, and VCRome2_1) or whole genome sequenced according to standard protocols.

### IGM bioinformatics pipeline

After quality filtering the raw sequence data using CASAVA (Illumina, Inc., San Diego, CA), the Illumina lane-level FASTQ files were aligned to the Human Reference Genome (NCBI Build37/hg19) using the Burrows-Wheeler Alignment Tool (BWA).[20] Picard (http://picard.sourceforge.net) was used to remove duplicate reads and process these lane-level SAM files, resulting in a sample-level BAM file that was used for variant calling. Variant and genotype calling was performed using the GATK software with local re-alignment around insertion/deletion variants and base quality recalibration for variants[21].

Variants for analysis were restricted to the consensus coding sequence public transcripts (CCDS release 14) plus 2 base pair intronic extensions[22]. Variants were further required to have: i) at least 10-fold coverage, ii) quality score (QUAL) of at least 30, iii) genotype quality (GQ) score of at least 20, iv) quality by depth (QD) score of at least 2, v) mapping quality (MQ) score of at least 40, vi) read position rank sum (RPRS) score greater than -3, vii) mapping quality rank sum (MQRS) score greater than -6, viii) indels were required to have a maximum Fisher’s strand bias (FS) of 200, ix) variants were screened according to VQSR tranche calculated using the known SNV sites from HapMap v3.3, dbSNP, and the Omni chip array from the 1000 Genomes Project to “PASS” SNVs were required to achieve a tranche of 99.9% for SNVs in genomes and exomes and 99% for indels in genomes, x) for heterozygous genotypes, the alternate allele ratio was required to be ≥25%. Finally, variants were excluded if they were among a predefined list of known sequencing artifacts or if they were marked by EVS (http://evs.gs.washington.edu/EVS/)[23] or ExAC (http://exac.broadinstitute.org/about)[16] as being problematic variants. Variants were annotated to Ensembl 73[24] using SnpEff[25].

### Quality control, relatedness check and principal component analysis

Any exomes with gender discordance between clinically-reported and X:Y coverage ratios were removed, as were contaminated samples according to VerifyBamID[26].

Before running gene-based collapsing analysis, we implemented both sample- and site-level pruning procedures to minimize the systemic bias in data that might lead to spurious association or reduced power to detect real association. The site-pruning procedure (coverage harmonization) is described in the section below. Here, we described the sample-level pruning procedure including removing related individuals and population outliers identified in principal component analysis (PCA).

To identify related individuals, we generated genotype data in PLINK format[27] and then used KING[28] to calculate pairwise kinship coefficients for all case and control subjects. We used the kinship coefficient 0.1 as a cutoff and removed samples introducing relatedness while preferentially retaining cases; we retained samples with a higher overall coverage in the CCDS regions to break ties if applicable. After this step, 492 of the 496 cases and 12,248 of the 12,916 controls were kept for further analysis.

Next we ran PCA using EIGENSTRAT[29] on the 492 cases and 12,248 controls with a LD-pruned (r^2^ threshold 0.1) list of single-nucleotide polymorphisms (SNPs) extracted from exomic sequencing data. After removing outliers given a sigma threshold (6.0 along the top10 principal components) for 5 iterations, a total of 488 cases and 12,151 controls entered gene-based collapsing analysis (S1 Fig).

### Coverage harmonization (site-pruning)

For the 488 cases and 12,151 controls entering association analysis, at least 10-fold coverage was achieved for an average of 93.20% in cases and 95.19% in controls of the 33.27 MB of the consensus coding sequence (CCDS release 14) plus 2 base pair (bp) intronic extensions (to accommodate canonical splice site variants). To address the confounding effect introduced by imbalance of coverage between cases and controls, we pruned out sites with uneven coverage in cases and controls using our previously described site-pruning procedure[30]. Specifically, for each site in CCDS plus 2 bp extensions, we determined the percentages of cases and controls that had at least 10-fold coverage, and that site was excluded from further analysis if the percentages differed by >11.97% between cases and controls. This site-pruning procedure removed 8.58% of the CCDS (+2bp intronic extensions) bases from the analysis. After site pruning, at least 10-fold coverage was achieved for an average of 88.27% in cases and 88.12% in controls of the 33.27 MB CCDS (+2bp intronic extensions) bases. These sites entered the association analysis where case and control populations had a comparable coverage to accurately compare patterns of variation gene by gene.

### Collapsing analysis

To identify genes associated with EE under the case-control association analysis framework, we performed a genome-wide search for an enrichment of “qualifying variants” in protein-coding genes in cases compared to controls looking for risk alleles. A “qualifying variant” was determined by a set of criteria, based on allele frequency and functional predictions, designed to capture the characteristics of pathogenic variants associated with EE. Specifically, in this study, we focused on “ultra-rare”, highly impactful variants, and a variant was determined to be qualifying if it: 1) was absent in the Exome Variant Server (EVS) and Exome Aggregate Consortium (ExAC release 0.3); 2) had ≤4 copies of variant allele in the 488 cases plus 12,151 controls; and 3) was predicted to be loss-of-function (stop_gained, frame_shift, splice_site_acceptor, splice_site_donor, start_lost, or exon_deleted) or missense “probably damaging” by PolyPhen-2 (HumDiv). We focused on this subset in an effort to try to capture the *de novo* variant signal that has been previously reported to play a role in a range of epilepsies and in particular EE subtypes [2, 31, 32]. For each gene, an indicator variable (1/0 states) was assigned to each individual based on the presence of at least one qualifying variant in the gene (state 1) or no qualifying variant in that gene (state 0); this was equivalent to a dominant genetic model. Accordingly, for a given gene, a qualifying case (or control) was defined to be a case (or control) subject carrying at least one qualifying variant in that gene. We used two-tailed Fisher’s exact test to evaluate statistical significance of genic association. To address the potential confounding effect of background rate of “qualifying variants,” we further constructed a logistic regression model including the total number of “ultra-rare” (absent in EVS and ExAC and having ≤4 copies of variant allele in the 488 cases plus 12,151 controls) synonymous variants per individual as covariate. To account for bias due to small counts of qualifying variant, we employed a Firth correction with profile likelihood based tests [33, 34]. With 18,668 CCDS genes we aimed to test, we adopted the genome-wide significance level of p = 2.68×10^−6^ using Bonferroni correction (0.05/18,668).

### Quantile-quantile probability plots and genomic inflation factor (λ)

Quantile-quantile plots were generated using a permutation-based expected probabilities distribution. To achieve this, for each model (matrix) we randomly permuted the case and control labels of the original configuration: 488 cases and 12,151 controls and then recomputed the Fisher’s Exact test for all genes. This was repeated 1,000 times. For each of the 1,000 permutations we ordered the p-values and then took the mean of each rank-ordered estimate across the 1,000 permutations, i.e., the average 1st order statistic, the average 2nd order statistic, etc. These then represent the empirical estimates of the expected ordered p-values (expected -log10(p-values)). This empirical-based expected p-value distribution no longer depends on an assumption that the p-values are uniformly distributed under the null. For comparison we have provide QQ plots for the actual p-values (S2 Fig) and empirically-based expected p-value distribution (S3 Fig).

To compute the permutation-based expected p-value distribution for Firth logistic regression, due to the presence of the covariate (the total number of “ultra-rare” synonymous variants per individual), we implemented permutation using the R package “BiasedUrn” (https://cran.r-project.org/web/packages/BiasedUrn/) to maintain the confounding role of covariate in each permuted data set while the association between genotype and disease was broken[35]. Permutation was performed 1,000 times and the empirical-based expected p-value distribution was calculated in the same way as described above. For comparison to the BiasedUrn permuted p-values, we have provided QQ plots for the actual p-values generated from the Firth logistic regression (S4 Fig).

## Supporting information

S1 Table Genome-wide gene-level analysis results. (XLSX)

S2 Table Qualifying variants (n = 73) in the 25 known EE genes identified in the 64 cases with trio WES data. (XLSX)

S3 Table IRB and approval numbers. (DOCX)

S4 Table 488 cases analyzed in gene-based collapsing analysis. (XLSX)

S1 Fig Principal component plots for 488 cases and 12,151 controls. Top: PC1 vs. PC2. Middle: PC2 vs. PC3. Bottom: PC1 vs. PC3.
(PDF)

S2 Fig Quantile-quantile (QQ) plot for genome-wide gene-based collapsing analysis using Fisher’s exact test based on actual p-value distribution. (PDF)

S3 Fig Quantile-quantile (QQ) plot for genome-wide gene-based collapsing analysis using Fisher’s exact test based on an empirical-based expected p-value distribution. (PDF)

S4 Fig Quantile-quantile (QQ) plot for genome-wide gene-based collapsing analysis using Firth logistic regression test based on actual p-value distribution. (PDF)
