# Supplementary material for: A case-control collapsing analysis identifies epilepsy genes implicated in trio sequencing studies focused on de novo mutations
Source: PLoS Genet. 2017 Nov 29;13(11):e1007104. doi: 10.1371/journal.pgen.1007104 (PMC5724893; doi:10.1371/journal.pgen.1007104)
Supplement: S3 Table — (DOCX) [file pgen.1007104.s003.docx]

**S3 Table.** IRB and approval numbers

The Saint Barnabas Medical Center Institutional Review Board (# 05-32)

The Committee on Clinical Investigation at Children’s Hospital Boston (# 09-05-0234, #05-05-076R, X10-04-0197, # P00014659)

CHOP Institutional Review Board (# IRB 06-004997_AM7, #IRB 14-011186)

University of Pittsburg Institutional Review Board, Committee H (REN12040010 / PRO10020513)

Cincinnati Children’s Hospital Med Ctr Institutional Review Board (# 2008-0643)

Colorado Multiple Institutional Review Board at University of Colorado (#09-0871)

The Einstein Institutional Review Board (#05-07-193S)

Emory Institutional Review Board (#IRB00024900)

Committee for the Protection of Human Subjects at The University of Texas (#HSC-MS-09-0670)

Office of Human Subjects Research at The Johns Hopkins University (#NA_00016067)

Mayo Clinic Institutional Review Board (# PR07-005798-04)

Austin Health Human Research Ethics Committee (H2009 / 03646)

Institutional Review Board NYU School of Medicine (#11846)

Institutional Review Board at Rush University Medical Center (#L05040705-IRB01-AM01)

Seattle Children’s Institutional Review Board (#13142)

Institutional Review Board at University of Alabama at Birmingham (#0990-0263)

Committee on Human Research at University of California San Francisco (10-03782, 14-14356)

IRBMED at University of Michigan(#IRB00001995)

Vanderbilt University Institutional Review Board (#100531)

University of Virginia Institutional Review Board for Health Sciences Research (#15126)

Washington University in St.Louis Human Research Protection Office (#10-0600)

Ethical Committee University Hospital Antwerp (# 13/21/229)

Columbia University Medical Center (# IRB-AAAO5656)

New York University IRB (# i14-01487)

Lurie Childrens Hospital IRB (IRB 2015-262_

Paediatric Ethic Committee of the Tuscany Region’ (#2014-0000559)
