# Supplementary figures and images for: A case-control collapsing analysis identifies epilepsy genes implicated in trio sequencing studies focused on de novo mutations
Source: PLoS Genet. 2017 Nov 29;13(11):e1007104. doi: 10.1371/journal.pgen.1007104 (PMC5724893; doi:10.1371/journal.pgen.1007104)

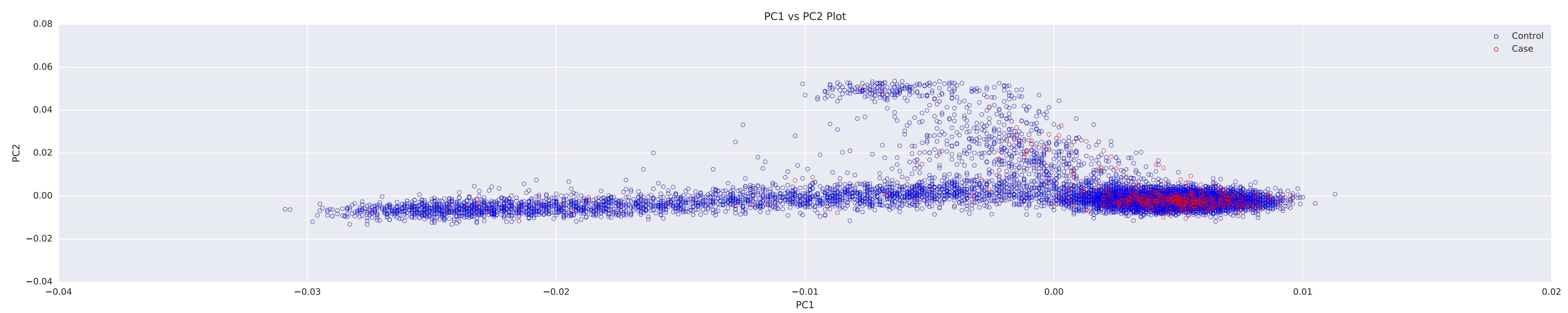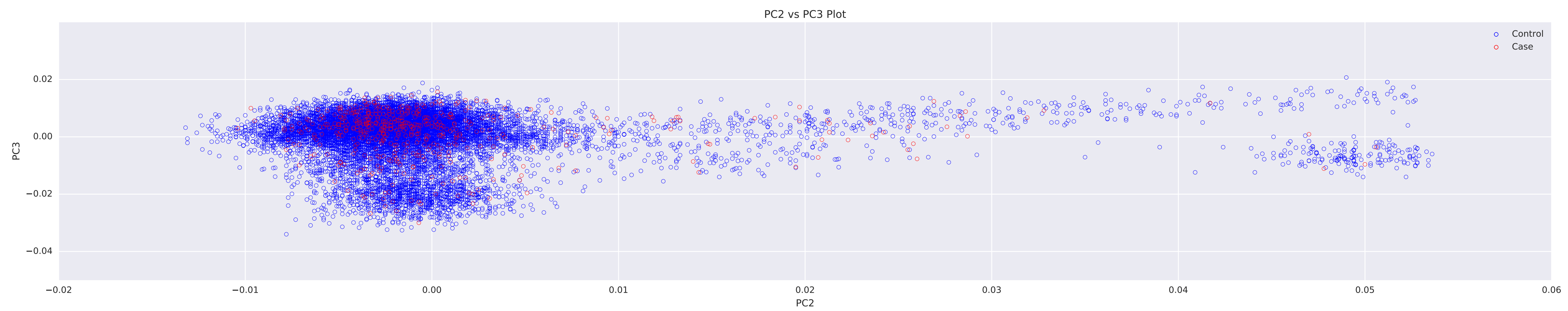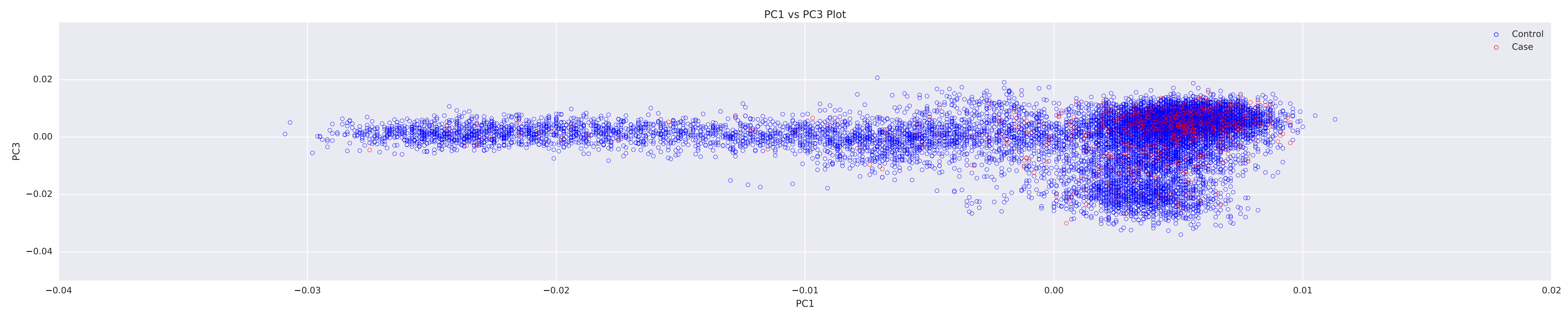

Supplement: S1 Fig — Top: PC1 vs. PC2. Middle: PC2 vs. PC3. Bottom: PC1 vs. PC3. (PDF) [file pgen.1007104.s005.pdf]

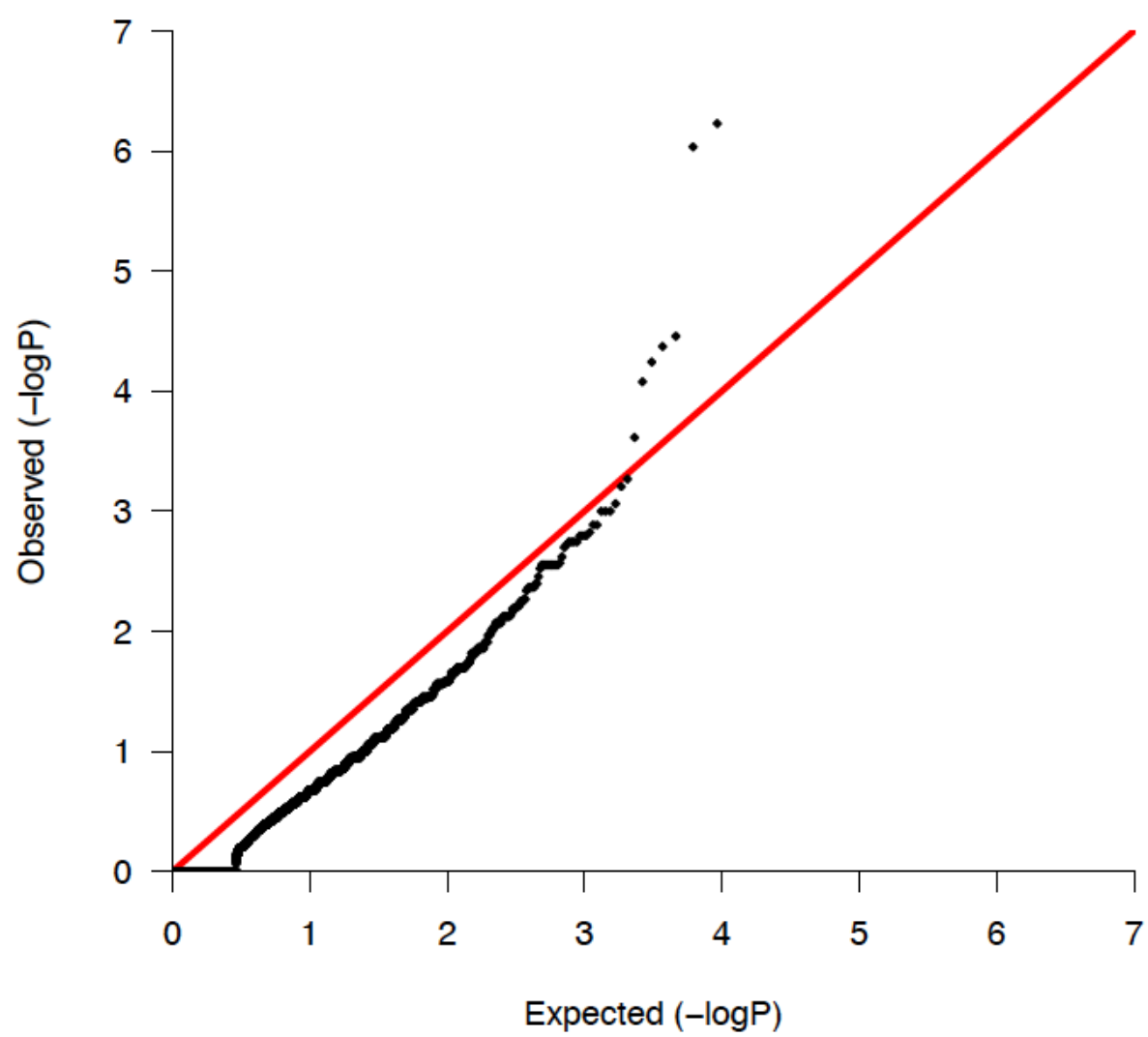

Supplement: S2 Fig — (PDF) [file pgen.1007104.s006.pdf]

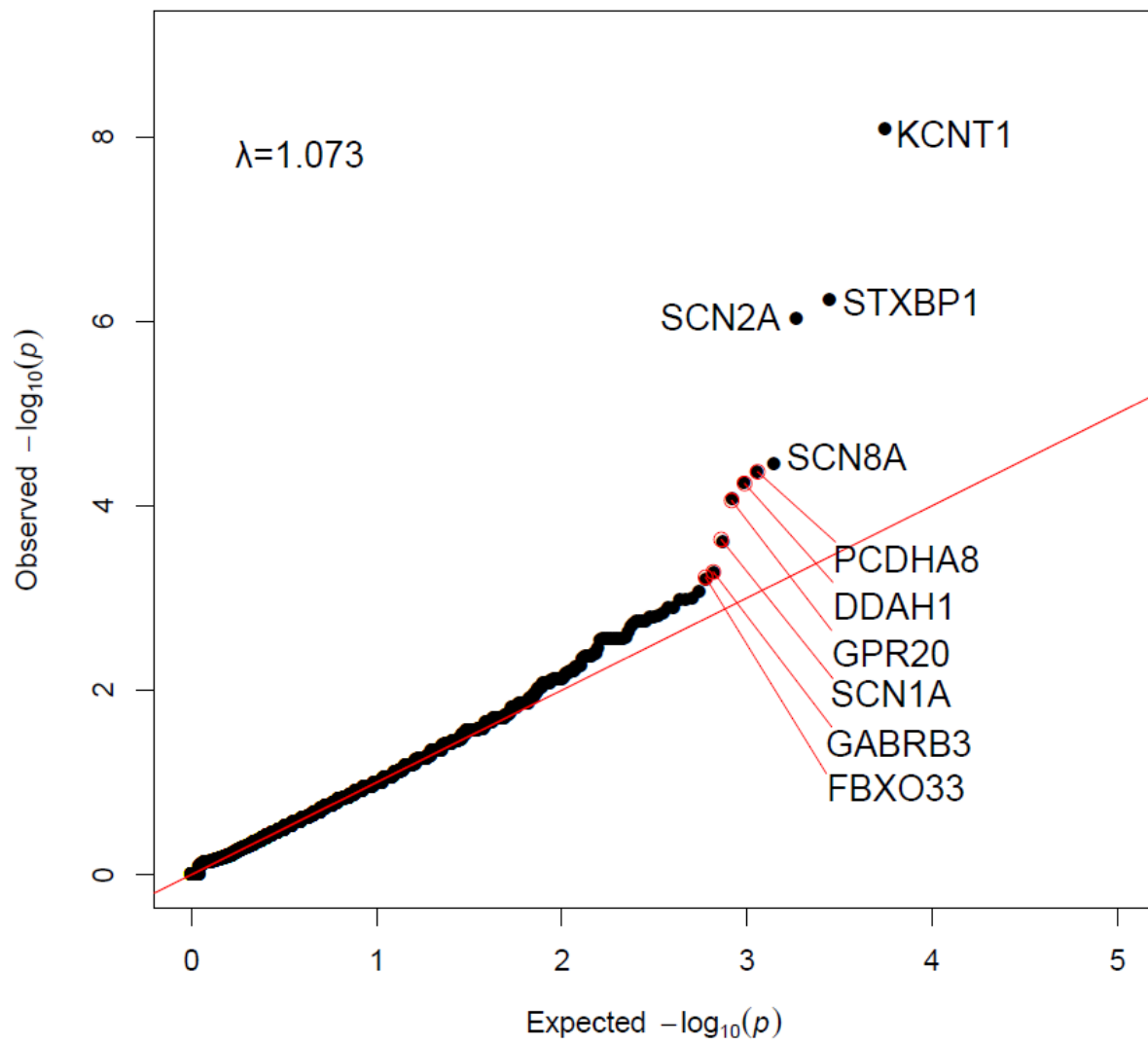

Supplement: S3 Fig — (PDF) [file pgen.1007104.s007.pdf]

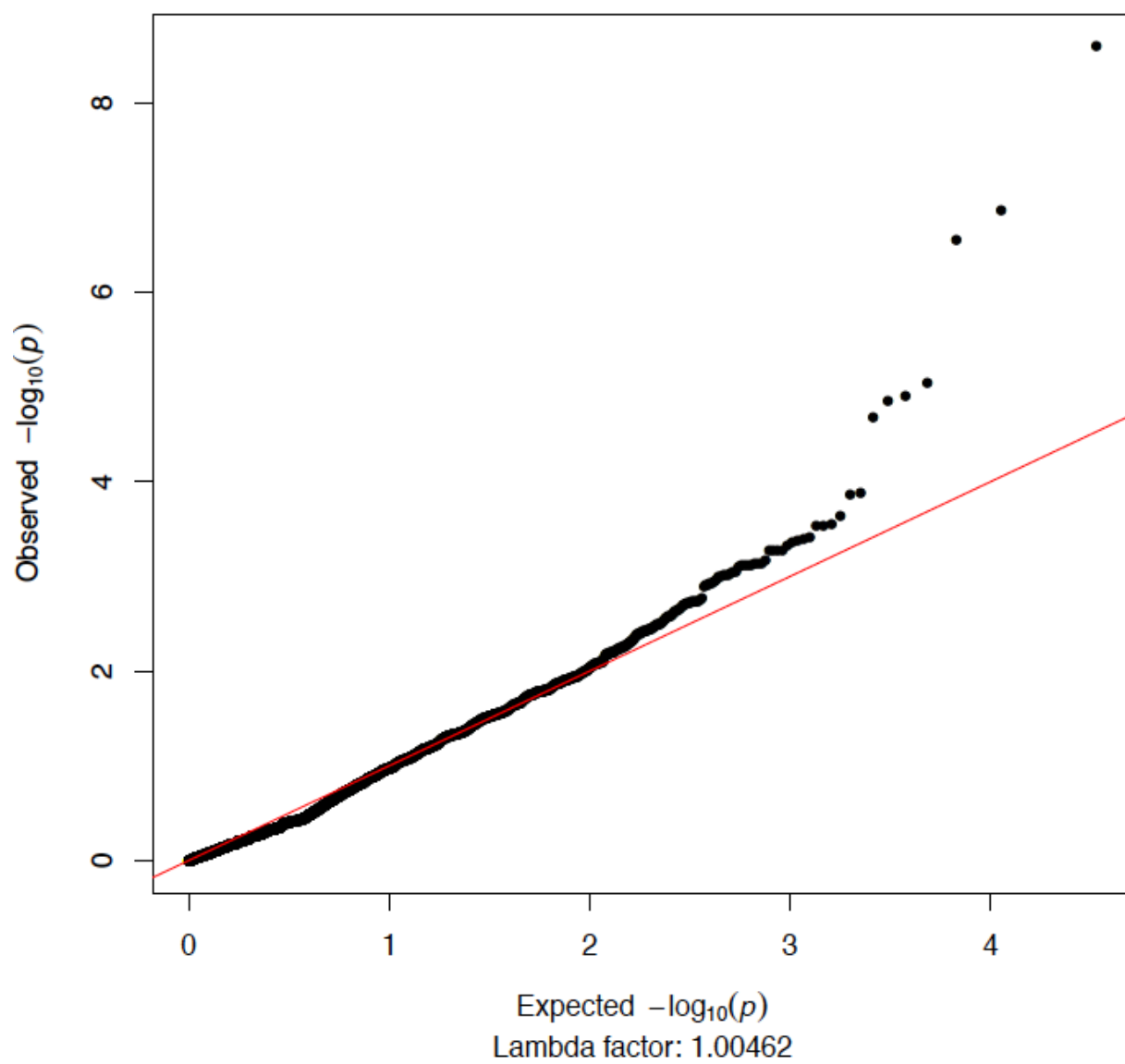

Supplement: S4 Fig — (PDF) [file pgen.1007104.s008.pdf]
